# Predictors of cognitive impairment in newly diagnosed Parkinson’s disease with normal cognition at baseline: A 5-year cohort study

**DOI:** 10.3389/fnagi.2023.1142558

**Published:** 2023-02-28

**Authors:** Jing Chen, Danhua Zhao, Qi Wang, Junyi Chen, Chaobo Bai, Yuan Li, Xintong Guo, Baoyu Chen, Lin Zhang, Junliang Yuan

**Affiliations:** ^1^Department of Neurology, Peking University Sixth Hospital, Peking University Institute of Mental Health, NHC Key Laboratory of Mental Health (Peking University), National Clinical Research Center for Mental Disorders (Peking University Sixth Hospital), Peking University, Beijing, China; ^2^Department of Neurology and Neurological Surgery, UC Davis Deep Brain Stimulation (DBS), Sacramento, CA, United States

**Keywords:** Parkinson’s disease, mild cognitive impairment, dementia, Montreal cognitive assessment, neuropsychological test

## Abstract

**Background and objective:**

Cognitive impairment (CI) is a substantial contributor to the disability associated with Parkinson’s disease (PD). We aimed to assess the clinical features and explore the underlying biomarkers as predictors of CI in patients with newly diagnosed PD (NDPD; less than 2 years).

**Methods:**

We evaluated the cognitive function status using the Montreal Cognitive Assessment (MoCA) and a battery of neuropsychological tests at baseline and subsequent annual follow-up for 5 years from the Parkinson’s Progression Markers Initiative (PPMI) database. We assessed the baseline clinical features, apolipoprotein (*APO*) *E* status, β-glucocerebrosidase (*GBA*) mutation status, cerebrospinal fluid findings, and dopamine transporter imaging results. Using a diagnosis of CI (combined mild cognitive impairment and dementia) developed during the 5-year follow-up as outcome measures, we assessed the predictive values of baseline clinical variables and biomarkers. We also constructed a predictive model for the diagnosis of CI using logistic regression analysis.

**Results:**

A total of 409 patients with NDPD with 5-year follow-up were enrolled, 232 with normal cognitive function at baseline, and 94 patients developed CI during the 5-year follow-up. In multivariate analyses, age, current diagnosis of hypertension, baseline MoCA scores, Movement disorder society Unified PD Rating Scale part III (MDS-UPDRS III) scores, and *APOE* status were associated with the development of CI. Predictive accuracy of CI using age alone improved by the addition of clinical variables and biomarkers (current diagnosis of hypertension, baseline MoCA scores, and MDS-UPDRS III scores, *APOE* status; *AUC* 0.80 [95% *CI* 0.74–0.86] vs. 0.71 [0.64–0.77], *p* = 0.008). Cognitive domains that had higher frequencies of impairment were found in verbal memory (12.6 vs. 16.8%) and attention/processing speed (12.7 vs. 16.9%), however, no significant difference in the prevalence of CI at annual follow-up was found during the 5-year follow-up in NDPD patients.

**Conclusion:**

In NDPD, the development of CI during the 5-year follow-up can be predicted with good accuracy using a model combining age, current diagnosis of hypertension, baseline MoCA scores, MDS-UPDRS III scores, and *APOE* status. Our study underscores the need for the earlier identification of CI in NDPD patients in our clinical practice.

## Introduction

Parkinson’s disease (PD) is the second-most common neurodegenerative disorder, that affects 2–3% of the population ≥ 65 years, with a prevalence set to double by 2030 (Poewe et al., 2017; Aarsland et al., 2021). Dementia occurs in at least 75% of patients who have had PD for more than 10 years (Aarsland and Kurz, 2010). Cognitive impairment (CI) can potentially occur at different stages (Aarsland et al., 2001), severely affect the quality of life and function, and increase caregiver burden and health-related costs (Aarsland et al., 2021). As the focus has been on early cognitive changes among PD patients, the course of mild cognitive impairment (MCI) can be quite variable. Given that PD patients who revert from MCI to normal cognition have an increased long-term risk for dementia (Pedersen et al., 2017; Jones et al., 2018), earlier risk factor stratification for CI could help to prognosticate the disease course and appropriate interventions in the early PD population.

The prior studies reported that some risk factors were related to CI in patients with PD. A recent meta-analysis suggested that the following variables were independently associated with the future development of MCI or dementia: the presence of hallucinations, older age, the overall severity of motor symptoms, presence of speech impairment, older age at onset, bradykinesia severity, higher Hoehn and Yahr stage, axial impairment, a low level of education, presence of depression, and male sex (Marinus et al., 2018). Studies have shown that current diagnoses of diabetes mellitus and hypertension were two important modifiable predictors of cognitive decline in PD (Mollenhauer et al., 2019; Nicoletti et al., 2021; Athauda et al., 2022). Baseline global cognitive function, hyposmia, rapid eye movement (REM) sleep behavior disorder (RBD), dysautonomia, apolipoprotein (*APO*) *E* status, β-glucocerebrosidase gene (*GBA*) status, and dopamine deficit on dopamine transporter (DAT)-imaging have all been suggested as predictors of MCI or dementia in patients with PD (Hu et al., 2014; Mata et al., 2014; Liu et al., 2017; Schrag et al., 2017; Leta et al., 2021; Barrio et al., 2022; Dijkstra et al., 2022). Varieties of studies have explored the association of serum uric acid and cerebral spinal fluid (CSF) findings, however, the results were still controversial (Liu et al., 2015; Pellecchia et al., 2016; Terrelonge et al., 2016; Johar et al., 2017; Seifar et al., 2022). The stage and duration of disease varied among participants in those studies, most of the studies included PD patients with CI at baseline, and some studies used healthy people as control. As a result, the above findings may not apply to newly diagnosed PD (NDPD) patients with normal cognition at baseline.

In our present study, we will select NDPD patients (diagnosed with PD for 2 years or less at screening visit) with normal cognition at baseline and evaluate the contribution of those risk factors to predict the development of CI (combining MCI and dementia) during the 5-year follow-up. Our study provides a correlation between specific risk factors and the onset of CI leading to improvement in the management of dementia in NDPD.

## Materials and methods

### Study design and participants

In this cohort study, we investigated the clinical and biomarker predictors and clinical characteristics of CI in NDPD (followed up for 5 years) using data from the Parkinson’s Progression Markers Initiative (PPMI).^1^ The PPMI is an international, multi-center, longitudinal, observational study aiming to identify biomarkers of PD progression in *de novo* PD patients (diagnosed within 2 years). Details of the PPMI eligibility criteria are given on the PPMI website. Written informed consent was provided by each PPMI participant, and the PPMI study was approved by the institutional board at each study site.

The data were downloaded from the PPMI website on August 1, 2022. Firstly, we included PD patients meeting the following criteria to investigate the predictors of CI: (1) with annual follow-ups for 5 years; (2) MoCA scores at baseline and 5-year follow-up were available; and (3) cognitive function at baseline was normal. PD patients who followed for less than 5 years were excluded. Then the included PD patients were divided into those with CI and those without CI based on whether they had CI at the annual follow-up for 5 years. Secondly, we included all the PD patients with annual follow-ups for 5 years to assess their cognitive performance at each visit. And the prevalence of CI at baseline and 5-year cumulative incidence of CI were calculated. Thirdly, PD patients with MoCA scores at baseline and 5-year follow-up available were included. The global cognitive function fluctuation in the early stage of PD was evaluated by changes in MoCA scores at baseline and 5-year follow-up.

### Outcomes

The outcome was defined as CI (combining MCI and dementia). Global cognitive function was assessed by MoCA, with suggested cutoffs of < 26/30 for MCI and < 21/30 for dementia by Movement Disorder Society MCI task force level guidelines (Emre et al., 2007; Dalrymple-Alford et al., 2010; Litvan et al., 2012). A diagnosis of MCI required that cognitive deficits are not sufficient to interfere significantly with functional independence, although subtle difficulties in complex functional tasks may be present (Litvan et al., 2012). A diagnosis of PD dementia (PDD) required evidence that the functional impairment caused by CI is sufficient to interfere with activities of daily living (Emre et al., 2007). The cognitive domains were assessed by a battery of neuropsychological tests, which included the Hopkins Verbal Learning Test (HVLT) total recall and HVLT recognition discrimination for verbal memory, Benton Judgment of Line Orientation (JOLO) for visuospatial function, Letter-Number Sequencing (LNS) and the Semantic (animal) fluency Test (SFT) for executive function/working memory, and the Symbol-Digit Modalities Test (SDMT) for attention/processing speed. Based on the impairment of cognitive domains, MCI was defined according to the PPMI protocol and the Movement Disorder Society MCI task force level I guidelines (Litvan et al., 2012). PD patients with MCI (PD-MCI) were defined as scores on two or more of the HVLT total recall, HVLT recognition discrimination, JOLO, LNS, SFT, and SDMT were more than 1.5 standard deviations below normal, with no functional impairment due to CI. These criteria have been applied and validated in several studies (Schrag et al., 2017; Chen et al., 2021).

### Candidate predictors

Clinical variables included in our study were the age of onset, sex, years of education, disease duration, current diagnosis of diabetes mellitus, and hypertension. PD motor severity was measured by the MDS-UPDRS part III (MDS-UPDRS III), the Hoehn and Yahr stage. Axial impairment was assessed by tremor dominant, postural instability/gait difficulty, or indeterminate phenotypes of PD calculated with the use of published methods (Stebbins et al., 2013). The presence of speech impairment was considered dichotomous variable depending on the sum of item 2.1 and 3.1 of the MDS-UPDRS scored 0 vs. ≥ 1. PD non motor symptoms (NMS) were assessed by the University of Pennsylvania Smell Identification Test (UPSIT) for sense of smell, the 15-item Geriatric Depression Scale (GDS) for depression, the Scale for Outcomes in Parkinson’s disease for Autonomic symptoms (SCOPA-AUT) for dysautonomia, and the question 6 of RBD Screening Questionnaire (RBDSQ-q6) for probable RBD (pRBD; Schrag et al., 2017). Neurological orthostatic hypotension (nOH; Norcliffe-Kaufmann et al., 2018) was also used to assess autonomic function. The presence of psychosis was considered dichotomous variable depending on item 1.2 of the MDS-UPDRS scored 0 vs. ≥ 1. The presence of apathy was considered dichotomous variables depending on item 1.5 of the MDS-UPDRS scored 0 vs. ≥ 1. For biomarker studies, we included serum uric acid, *APOE* ε4 status (ε4 homozygous, heterozygous, or negative), *GBA* mutation status, and DAT imaging data for mean caudate and putaminal uptake relative to uptake in the occipital area, and asymmetry of caudate and putaminal uptake (side with the highest divided by side with the lowest uptake; Schrag et al., 2017). We evaluated CSF for α-synuclein, Aβ_42_, total tau (t-tau), phosphorylated tau_181_ (p-tau), and the calculated ratio of Aβ_42_ to t-tau.

### Statistical analysis

We compared groups using *χ*^2^ tests for categorical data, student *t* tests for normally distributed variables, and Mann–Whitney tests for non-parametric data. Univariate logistic regression analysis was used to identify possible risk factors for CI developed during the 5-year follow-up. Variables with values of *p* < 0.05 in univariate logistic regression analysis and no high correlation (*r* > 0.5) with each other were included in a multivariate model. If there was a correlation between variables, the one with a smaller *p* value was selected. Variables were removed from the multivariate model with the backward selection method until all variables were significant at *p* < 0.05. Receiver operating characteristic curves were drawn and areas under the curve were calculated to estimate the prediction accuracy. Statistical analysis was carried out with SPSS version 26, and *p* < 0.05 was considered significant.

## Results

**Figure 1:**
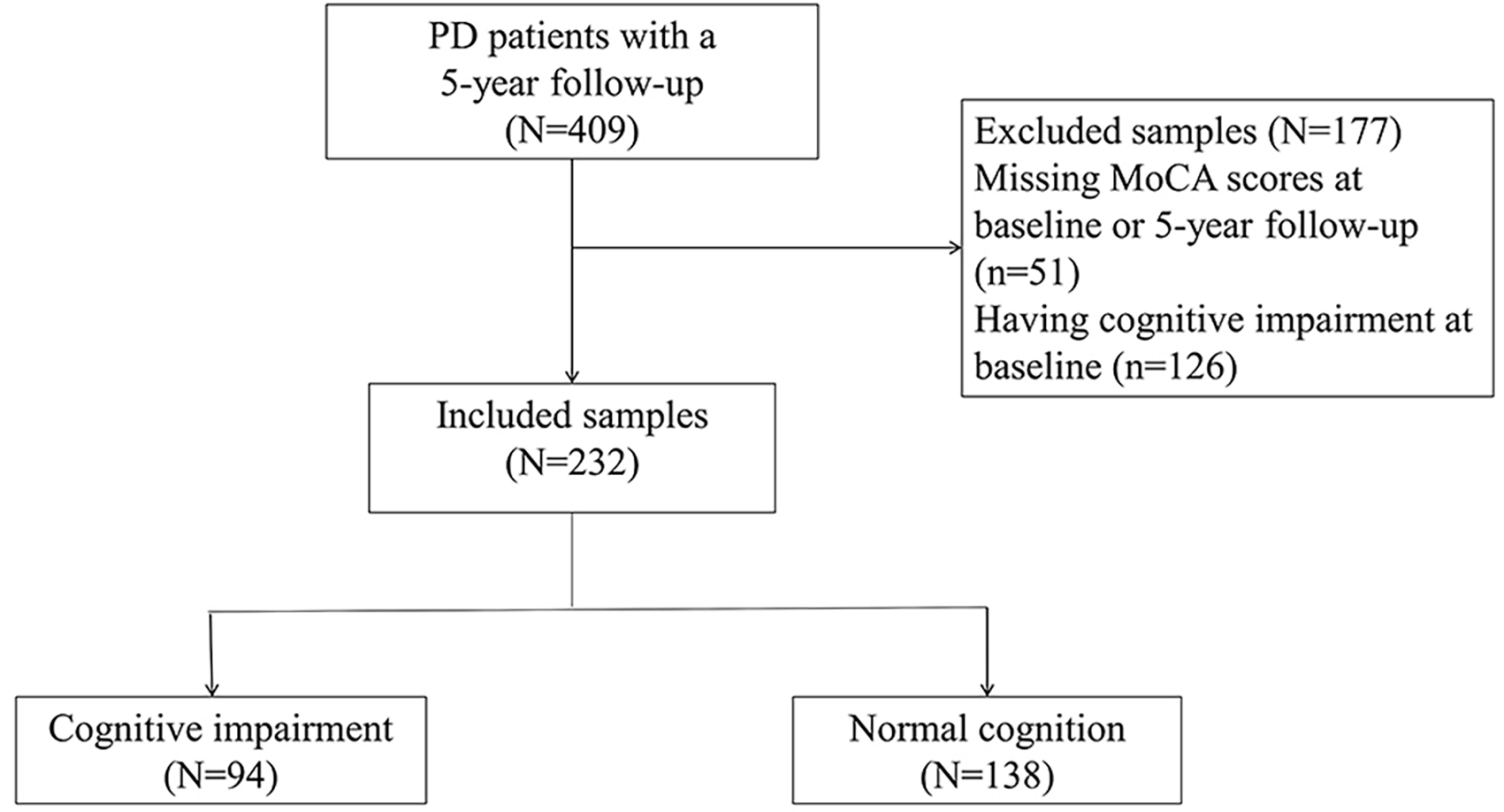
Flowchart of patient selection and classification.

There were 409 NDPD patients with a minimum 5-year follow-up in the PPMI database. 51 cases did not have recorded MoCA scores at baseline or 5-year follow-up and 126 cases diagnosed with MCI at baseline. These cases were excluded. A total of 232 subjects were included to investigate predictors of CI, of whom 94 met the CI criteria during the 5-year follow-up (Figure 1). Baseline clinical characteristics and biomarkers of the patients with or without CI were shown in Table 1. At baseline, NDPD patients with CI were older and they had a higher proportion of male sex, current diagnosis of hypertension, Hoehn and Yahr stage 2, and *APOE* ε4 homozygotes than those without CI during the 5-year follow-up. Lower baseline MoCA and UPSIT scores and higher SCOPA-AUT gastrointestinal domain, RBDSQ question 6, and MDS-UPDRS III scores were observed in NDPD patients with CI (Table 1). No significant difference was found in *GBA* mutation status, CSF findings, and DAT biomarkers between the two groups.

**Table 1:** Baseline characteristics and biomarkers of the patients with newly diagnosed Parkinson’s disease (less than 2 years) with or without cognitive impairment developed during the 5-year follow-up.

	Patients with cognitive impairment	Patients without cognitive impairment	*p* value^*^
**Demographic and clinical characteristics**			
Age, years	63.4 (9.1)	57.2 (9.3)	0.000
Men	66 (70%)	76 (55%)	0.020^†^
Education, years	15.6 (3.3)	15.8 (3.3)	0.788
Diabetes mellitus	2 (2.1%)	10 (4.3%)	0.208^†^
Hypertension	42 (18.1%)	10 (7.2%)	0.000^†^
Disease duration, months	4 (2–10)	5 (2–15)	0.371^‡^
MoCA score	27 (27–29)	29 (28–30)	0.000^‡^
UPSIT score	20.9 (8.6)	24.1 (8.2)	0.005
GDS score	5 (5–6)	5 (5–6)	0.840^‡^
Psychosis	3 (3.2%)	5 (3.6%)	0.583^†^
Apathy	16 (17.0%)	19 (13.8%)	0.497^†^
SCOPA-AUT total score	10 (6–14)	8 (6–13)	0.102^‡^
	Gastrointestinal domain
2 (1–4)		1 (0–3)	0.021^‡^
Urinary-sexual domain	5 (2–7)	5 (2–7)	0.408^‡^
Cardiovascular domain	0 (0–1)	0 (0–1)	0.632^‡^
Thermoregulatory domain	1 (0–2)	1 (0–2)	0.522^‡^
Pupillomotor domain	0 (0–1)	0 (0–1)	0.198^‡^
nOH	24 (25.5%)	25 (18.4%)	0.193^†^
RBDSQ-q6	0 (0–1)	0 (0–1)	0.014^‡^
Speech impairment	53 (56.4%)	72 (52.2%)	0.528^†^
H & Y			
1	39 (41.9%)	80 (59.7%)	0.008^†^
2	54 (58.1%)	54 (40.3%)	
MDS-UPDRS motor score	19.5 (14–26)	16 (12–21)	0.003
Motor subtype			
Tremor-dominant	78 (84.8%)	106 (77.4%)	0.38^†^
Postural instability and gait difficulty	8 (8.7%)	17 (12.4%)	
Indeterminate	6 (6.7%)	14 (10.2%)	
**Uric acid, APOE, GBA, CSF, and DAT imaging markers**			
Uric acid	315 (85)	307 (81)	0.474
*APOE* ε4 status			
Negative	65 (69.1%)	112 (81.2%)	0.041^†^
Heterozygous	23 (24.5%)	24 (17.4%)	
Homozygous	6 (6.4%)	2 (1.4%)	
*GBA* mutation	17 (17.5%)	19 (13.9%)	0.445^†^
CSF findings, pg./ml			
α-synuclein (participants with hemoglobin < 200 pg./ml)	1826.1 (684.5)	1915.9 (884.6)	0.462
Aβ_42_	366.7 (96.3)	389.8 (95.9)	0.114
Total tau	43.2 (15.5)	42.9 (15.6)	0.895
Phosphorylated tau	17.0 (13.4)	16.2 (9.3)	0.634
Aβ_42_:*t*-tau ratio	9.1 (2.7)	10.0 (3.2)	0.056
DAT imaging (striatal binding ratio)			
Mean putaminal uptake	0.8 (0.3)	0.8 (0.3)	0.898
Mean caudate uptake	2.0 (0.5)	2.0 (0.5)	0.810
Putaminal asymmetry	1.5 (0.4)	1.6 (0.5)	0.074
Caudate asymmetry	1.2 (0.2)	1.2 (0.2)	0.498

^*^Student *t* tests. ^†^*χ*^2^ test. ^‡^Non-parametric statistics (median, IQR, Mann–Whitney test). Data are mean (SD), median (IQR), or *n* (%). MoCA = Montreal Cognitive Assessment. UPSIT = University of Pennsylvania Smell Inventory Test. GDS = Geriatric Depression Scale. SCOPA-AUT = The Scale for Outcomes in Parkinson’s disease for Autonomic symptoms. nOH = neurological orthostatic hypotension. RBDSQ q6 = Question 6 of Rapid Eye Movement Sleep Behavior Disorder Screening Questionnaire. H & Y = Hoehn and Yahr stage. MDS-UPDRS = Movement Disorder Society Unified Parkinson’s Disease Rating Scale. APOE = apolipoprotein E. GBA = β-glucocerebrosidase gene. DAT = dopamine transporter.

No data were missing for the age of onset, sex, years of education, medical history, disease duration, baseline MoCA scores, baseline results of the neuropsychologic tests, UPSIT scores, GDS scores, SCOPA-AUT scores, RBDSQ scores, MDS-UPDRS scores, *GBA* mutation status data, and the *APOE* status data. The results of the neuropsychological tests were missing for 14 patients at 1-year follow-up, for 20 patients at 2-year follow-up, for seven patients at 3-year follow-up, for 20 patients at 4-year follow-up, and for 21 patients at 5-year follow-up. The data was missing for two patients for Hoehn and Yahr stage information and 16 patients for serum uric acid. Baseline CSF findings were missing for Aβ_42_ and α-synuclein in 53 patients, for p-tau in 55 patients, and t-tau in 56 patients. The analyses were repeated by imputing missing predictor variable data with means (data not shown). These missing data did not change the overall results of any analysis.

**Figure 2:**
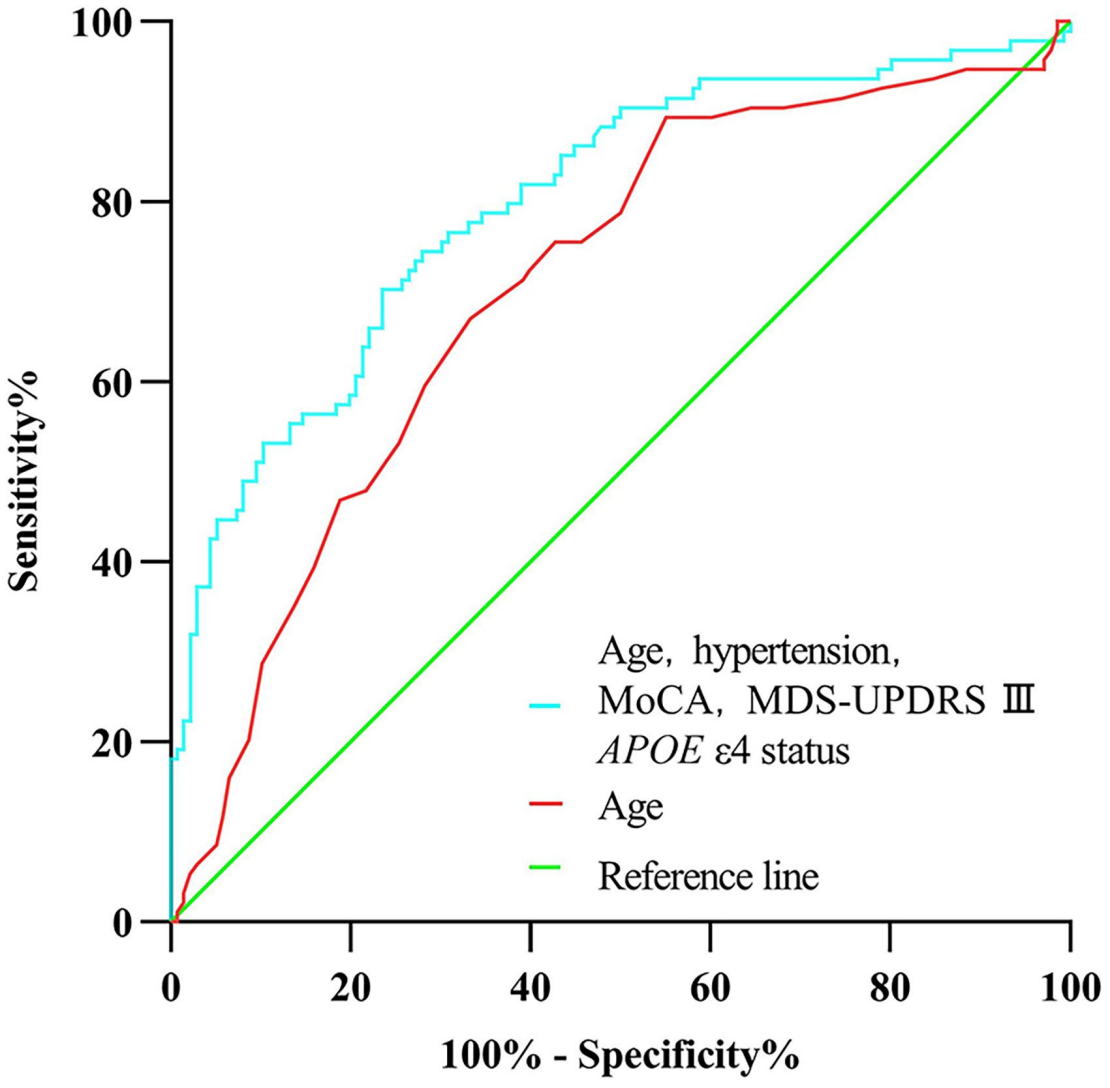
Receiver operating characteristic curve of prediction of cognitive impairment developed during the 5-year follow-up in newly diagnosed Parkinson’s disease patients (less than 2 years) with normal cognition at baseline.

In univariate analysis, the age of onset, sex, current diagnosis of hypertension, baseline MoCA scores, UPSIT scores, SCOPA-AUT gastrointestinal domain scores, RBDSQ-q6 scores, MDS-UPDRS III scores, the Hoehn and Yahr stage, and *APOE* status were associated with CI (Table 2). In multivariate analyses, CI was associated with the age of onset, current diagnosis of hypertension, baseline MoCA scores, MDS-UPDRS III scores, and *APOE* status (Table 2). In a logistic regression analysis with CI as the dependent variable, using the age of onset, current diagnosis of hypertension, baseline MoCA scores, MDS-UPDRS III scores, and *APOE* ε4 status as independent variables (Table 2), predictive accuracy was higher than for age alone (*AUC* 0.80 [95% *CI* 0.74–0.86] vs. 0.71 [0.64–0.77], *p* = 0.003; Figure 2).

**Table 2:** Univariate and multivariate logistic regression analyses show associations between risk factors and cognitive impairment in patients with newly diagnosed Parkinson’s disease (less than 2 years).

	Univariate analysis			Multivariate analysis		
Demographic and clinical characteristics	Coefficient	COR (95%CI)	*p* value	Coefficient	AOR (95%CI)	*p* value
Age, years	0.074	1.007 (1.043–1.111)	0.000^*^	0.063	1.065 (1.028–1.104)	0.001
Men	0.654	1.923 (1.104–3.349)	0.021^*^	.	.	.
Hypertension	1.114	3.045 (1.609–5.762)	0.001^*^	0.973	2.646 (1.271–5.512)	0.009
MoCA score	0.597	1.817 (1.443–2.289)	0.000*	0.511	1.667 (1.295–2.145)	0.000
UPSIT score	0.045	1.046 (1.013–1.08)	0.006^*^	.	.	.
SCOPA-AUT score Gastrointestinal domain	0.159	1.172 (1.043–1.318)	0.008^*^	.	.	.
	RBDSQ-q6	0.294	1.341 (1.012–1.778)	0.041^*^	.	.	.
H & Y						
1		Ref		Ref		
2	0.718	2.051 (1.198–3.511)	0.009	.	.	.
MDS-UPDRS motor score	0.053	1.005 (1.021–1.09)	0.002^*^	0.038	1.039 (1.001–1.078)	0.047
*APOE* ε4 status						
Negative		Ref		Ref		
Heterozygous	0.502	1.651 (0.863–3.158)	0.130	0.624	0.103 (0.882–3.950)	0.103
Homozygous	1.643	5.169 (1.014–26.363)	0.048^*^	2.683	14.630 (1.962–109.063)	0.009
Constant				−6.296	0.002	0.000

^*^Variables with values of *p* < 0.05 in the univariate logistic regression analysis and no high correlation (*r* > 0.5) with each other were included in a multivariate model. MoCA = Montreal Cognitive Assessment. UPSIT = University of Pennsylvania Smell Inventory Test. SCOPA-AUT = The Scale for Outcomes in Parkinson’s disease for Autonomic symptoms. RBDSQ q6 = Question 6 of Rapid Eye Movement Sleep Behavior Disorder Screening Questionnaire. H & Y = Hoehn and Yahr stage. MDS-UPDRS III = Movement Disorder Society Unified Parkinson’s Disease Rating Scale (Part III). APOE = apolipoprotein E. Ref = reference.

The prevalence of CI at baseline was 30.8%, and the 5-year cumulative incidence of CI was 40.5% in NDPD patients. The higher frequencies of impairment of cognitive domains were seen in verbal memory (12.6 vs. 16.8%) and attention/processing speed (12.7 vs. 16.9%; Table 3), the lower levels of impairment were seen in executive function/working memory and visuospatial function in the early stage of PD (Table 3). The cognitive fluctuation of 336 NDPD patients were shown in Table 4. Of the 71 subjects who scored 21–25 at baseline MoCA, seven (9.9%) subjects scored less than 21 and 37 (52.1%) subjects scored 26–30 at 5-year follow-up (Table 4).

**Table 3:** Cognitive performance of 409 patients with newly diagnosed Parkinson’s disease (less than 2 years) at baseline and subsequent annual follow-up.

Cognitive domain	Variable	% (*N*)						*p* value
		BL	1-year	2-year	3-year	4-year	5-year	
Global cognition	MoCA							
	30–26	75.4% (407)	69.0% (374)	69.2% (373)	69.0% (387)	71.5% (355)	71.2% (337)	0.138
	25–21	21.9% (407)	29.4% (374)	27.3% (373)	27.1% (387)	24.0% (355)	22.6% (337)	
	<21	3.2% (407)	3.2% (374)	3.5% (373)	3.9% (387)	4.5% (355)	6.2% (337)	
Attention/processing speed	Symbol digit modalities score	12.6% (406)	14.4% (374)	15.5% (373)	16.6% (385)	15.3% (346)	16.6% (307)	0.626
Memory	HVLT total recall	15.4% (408)	16.8% (375)	13.9% (373)	14.2% (387)	14.4% (346)	15.3% (307)	0.897
	HVLT recognition discrimination	14.8% (406)	16.9% (373)	11.0% (373)	12.7% (387)	14.2% (345)	16.0% (307)	0.234
Executive function/working memory	Semantic (animal) fluency total	3.7% (407)	5.6% (376)	7.0% (374)	6.0% (387)	4.6% (346)	7.8% (308)	0.652
	Letter number sequencing	4.2% (407)	2.9% (373)	4.3% (370)	4.9% (384)	4.1% (345)	3.9% (307)	0.813
Visuospatial function	Benton judgment of line	2.3% (406)	7.0% (373)	3.2% (371)	4.7% (387)	3.8% (346)	4.2% (306)	0.379

MoCA = Montreal Cognitive Assessment. HVLT = Hopkins Verbal Learning Test.

**Table 4:** Global cognitive function fluctuation in the early stage of newly diagnosed Parkinson’s disease (PD; less than 2 years) evaluated by changes in MoCA scores at baseline and 5-year follow-up.

MoCA score at baseline	*N* (%)	MoCA score at 5-year follow-up	*n* (%)
26–30	257 (76.5%)	26–30	207 (80.5%)
		21–25	40 (15.6%)
		< 21	10 (3.9%)
21–25	71 (21.1%)	26–30	37 (52.1%)
		21–25	27 (38.0%)
		< 21	7 (9.9%)
<21	8 (2.4%)	21–25	5 (62.5%)
		< 21	3 (37.5%)

MoCA = Montreal Cognitive Assessment.

## Discussion

In this study, we identified the predictors of CI developed during the 5-year follow-up in NDPD with normal cognition at baseline. Apart from older age, the strongest clinical predictors were current diagnosis of hypertension, lower baseline MoCA scores, *APOE* ε4 status, and to a lesser extent, higher baseline MDS-UPDRS III scores. We also find that the natural course of CI is variable during the 5-year follow-up in NDPD.

Previous studies have identified clinically relevant risk factors for CI and dementia in PD patients. In line with previous studies, we found that age of onset, current diagnosis of hypertension, baseline MoCA scores, and MDS-UPDRS III scores were independent predictors of CI. PD patients who developed CI during the 5-year follow-up had higher proportions of the male sex, hyposmia, dysautonomia, pRBD, and higher Hoehn and Yahr stage. Older age and hypertension are reliable predictors of CI in the general population, which are not unique to PD. Higher scores at baseline MoCA and MDS-UPDRS III, and higher Hoehn and Yahr stage usually indicate more serious pathology underlying PD, which is associated with an increased risk of CI. Hyposmia, constipation, and sleep disorders have also been reported to be associated with cognitive decline in the early stage of PD (Hu et al., 2014; Schrag et al., 2017; Leta et al., 2021). They are the earlier risk factors and prodromal features for non-motor symptoms (NMS) of PD due to the propagation of α-synuclein following caudo-rostral from the periphery to the central nervous system (Blesa et al., 2022). Besides, we also found some inconsistent data such as no difference between the two groups in education years, current diagnosis of diabetes mellitus, depression, psychosis, apathy, orthostatic hypotension, presence of speech impairment, and the motor subtype. In terms of education years, the reason may be that subjects in PPMI studies generally have long years of schooling. The inconsistency of the rest clinical variables is attributed to the disease duration and cognitive status of participants in different studies.

The occurrence and progression of CI in PD are associated with the *APOE* ε4 allele in the absence of other genetic variants at the genome-wide level (Iwaki et al., 2019; D'souza and Rajkumar, 2020; Tan et al., 2021; Real et al., 2022). In accordance with prior studies (Mata et al., 2014; Schrag et al., 2017), we found that homozygous of the *APOE* ε4 allele is a predictor of CI in NDPD with normal cognition at baseline. *GBA* mutations reduce glucocerebrosidase and lysosomal activities as independent risk factors for both PD and dementia with Lewy body and are associated with accelerated cognitive decline in PD (Cilia et al., 2016; Liu et al., 2016; Chia et al., 2021). Studies have shown that *GBA* mutations are reliable predictors of dementia in PD patients (Liu et al., 2017; Phongpreecha et al., 2020), but no difference was found between PD patients with normal cognition and MCI (Phongpreecha et al., 2020), similar to this early-stage study, which may be partially related to the fact that the *GBA* penetrance in PD patients increases with age (Anheim et al., 2012; Gan-Or et al., 2019).

Low levels of CSF Aβ_42_ are an independent predictor of cognitive decline in PD in previous studies (Johar et al., 2017). However, the associations of p-tau and t-tau concentrations with cognitive decline in PD patients were still controversial (Johar et al., 2017). Indeed, in one study of PPMI, low Aβ_42_ levels and mean caudate uptake in DAT imaging were associated with the occurrence of MCI or dementia at a 2-year follow-up (Schrag et al., 2017). However, no differences were found between PD patients with and without CI on the CSF Aβ_42_, t-tau, p-tau concentrations, Aβ_42_: t-tau ratio, and DAT biomarkers in our study. The inconsistencies in the findings of different studies could be attributed to differences in the participants and the intervals of follow-up. Our study only included PD patients with normal cognitive function at baseline, however, most of the previous studies included PD patients with CI at baseline to explore the risk factors of CI (Hall et al., 2015; Schrag et al., 2017). These different findings might reflect the different pathology of CI underlying in PD patients with normal cognitive function and PD patients with cognitive impairment at baseline.

Our study has some strengths. Firstly, participants included in this study are NDPD patients with normal cognition at baseline and followed up for 5 years. Secondly, the risk factors and neuropsychological tests were quite comprehensive, and such predictors in this model are obtained relatively easily in clinical practice. Thirdly, the different findings on biomarkers of CSF suggest that the underlying pathology of CI may be distinct in PD patients with normal cognitive function and PD patients with cognitive impairment at baseline.

There are also several limitations in our study. First, in the PPMI study, the MoCA scores and five neuropsychological tests covered four cognitive domains except for language was used to assess cognitive function. According to the MDS Task Force level II criteria (Litvan et al., 2012), some patients who met MCI criteria might be missed, which would affect the accuracy of results, although studies have supported the prognostic validity of the abbreviated MCI in PD criteria (Hoogland et al., 2019). Second, limited by the sample size and follow-up time, we did not subdivide the CI groups into MCI and PDD, nor did we analyze the conversion from MCI to dementia. Third, we did not analyze the effect of PD medication on cognitive function since the subjects in our study were not treated with medication at baseline, and such influencing factors should not be ignored. Fourth, in spite that we built such a prediction model, however, we did not validate the efficacy and feasibility of the model in different populations of PD patients such as from China. Therefore, a larger sample and much more comprehensive assessment, and prolonged follow-up will be required in a future study.

In summary, we explored the predictor model of the development of CI in NDPD during the 5-year follow-up. Our study may contribute to the early identification of CI in PD patients. In a future study, our study should be validated and a larger sample, much more comprehensive assessment, and longer follow-up time will be needed.

## Data availability statement

The data analyzed in this study is subject to the following licenses/restrictions: The data were sourced from the Parkinson’s Progression Markers Initiative (PPMI) database. The original contributions presented in the study are included in the article, further inquiries can be directed to the corresponding author. Requests to access these datasets should be directed to junliangyuan@bjmu.edu.cn.

## Ethics statement

The studies involving human participants were reviewed and approved by the PPMI study was approved by the institutional board at each study site. The patients/participants provided their written informed consent to participate in this study.

## Author contributions

JY and JiC contributed to the conception and design of the research. XG and JuC collected the data. JiC and CB contributed to the analysis and interpretation of the data. JiC: wrote the first draft of the manuscript. DZ, QW, YL, BC, LZ, and JY helped with the critical revision of the manuscript. All authors contributed to the article and approved the submitted version.

## Funding

This study was supported by the National Natural Science Foundation of China (82071552) and the Chinese Academy of Sciences Grant (JCTD-2021-06).

## Conflict of interest

The authors declare that the research was conducted in the absence of any commercial or financial relationships that could be construed as a potential conflict of interest.

## Publisher’s note

All claims expressed in this article are solely those of the authors and do not necessarily represent those of their affiliated organizations, or those of the publisher, the editors and the reviewers. Any product that may be evaluated in this article, or claim that may be made by its manufacturer, is not guaranteed or endorsed by the publisher.

## Footnotes

^1^ www.ppmi-info.org

## References

[ref1] Aarsland D. Andersen K. Larsen J. P. Lolk A. Nielsen H. Kragh-Sørensen P. (2001). Risk of dementia in Parkinson's disease: a community-based, prospective study. Neurology 56, 730–736. doi: 10.1212/WNL.56.6.730, PMID: 11274306

[ref2] Aarsland D. Batzu L. Halliday G. M. Geurtsen G. J. Ballard C. Ray Chaudhuri K.. (2021). Parkinson disease-associated cognitive impairment. Nat. Rev. Dis. Primers. 7:47. doi: 10.1038/s41572-021-00280-3, PMID: 34210995

[ref3] Aarsland D. Kurz M. W. (2010). The epidemiology of dementia associated with Parkinson disease. J. Neurol. Sci. 289, 18–22. doi: 10.1016/j.jns.2009.08.034 19733364

[ref4] Anheim M. Elbaz A. Lesage S. Durr A. Condroyer C. Viallet F.. (2012). Penetrance of Parkinson disease in glucocerebrosidase gene mutation carriers. Neurology 78, 417–420. doi: 10.1212/WNL.0b013e318245f476, PMID: 22282650

[ref5] Athauda D. Evans J. Wernick A. Virdi G. Choi M. L. Lawton M.. (2022). The impact of type 2 diabetes in Parkinson's disease. Mov. Disord. 37, 1612–1623. doi: 10.1002/mds.29122, PMID: 35699244 PMC9543753

[ref6] Barrio I. R. Miki Y. Jaunmuktane Z. T. Warner T. De Pablo-Fernandez E. (2022). Association between orthostatic hypotension and dementia in patients with Parkinson disease and multiple system atrophy. Neurology. doi: 10.1212/WNL.0000000000201659. [Epub ahead of print]., PMID: 36526431 PMC9990860

[ref7] Blesa J. Foffani G. Dehay B. Bezard E. Obeso J. A. (2022). Motor and non-motor circuit disturbances in early Parkinson disease: which happens first? Nature reviews. Neuroscience 23, 115–128. doi: 10.1038/s41583-021-00542-9, PMID: 34907352

[ref8] Chen F. Li Y. Ye G. Zhou L. Bian X. Liu J. (2021). Development and validation of a prognostic model for cognitive impairment in Parkinson's disease with REM sleep behavior disorder. Front. Aging Neurosci. 13:703158. doi: 10.3389/fnagi.2021.703158, PMID: 34322014 PMC8311737

[ref9] Chia R. Sabir M. S. Bandres-Ciga S. Saez-Atienzar S. Reynolds R. H. Gustavsson E.. (2021). Genome sequencing analysis identifies new loci associated with Lewy body dementia and provides insights into its genetic architecture. Nat. Genet. 53, 294–303. doi: 10.1038/s41588-021-00785-3, PMID: 33589841 PMC7946812

[ref10] Cilia R. Tunesi S. Marotta G. Cereda E. Siri C. Tesei S.. (2016). Survival and dementia in GBA-associated Parkinson's disease: the mutation matters. Ann. Neurol. 80, 662–673. doi: 10.1002/ana.24777, PMID: 27632223

[ref11] Dalrymple-Alford J. C. Macaskill M. R. Nakas C. T. Livingston L. Graham C. Crucian G. P.. (2010). The MoCA: well-suited screen for cognitive impairment in Parkinson disease. Neurology 75, 1717–1725. doi: 10.1212/WNL.0b013e3181fc29c9, PMID: 21060094

[ref12] Dijkstra F. De Volder I. Viaene M. Cras P. Crosiers D. (2022). Polysomnographic predictors of sleep, motor, and cognitive dysfunction progression in Parkinson's disease. Curr. Neurol. Neurosci. Rep. 22, 657–674. doi: 10.1007/s11910-022-01226-2, PMID: 35994190

[ref13] D'souza T. Rajkumar A. P. (2020). Systematic review of genetic variants associated with cognitive impairment and depressive symptoms in Parkinson's disease. Acta Neuropsychiatr. 32, 10–22. doi: 10.1017/neu.2019.28, PMID: 31292011

[ref14] Emre M. Aarsland D. Brown R. Burn D. J. Duyckaerts C. Mizuno Y.. (2007). Clinical diagnostic criteria for dementia associated with Parkinson's disease. Mov. Disord. 22, 1689–1707. doi: 10.1002/mds.21507 17542011

[ref15] Gan-Or Z. Alcalay R. N. Makarious M. B. Scholz S. W. Blauwendraat C. (2019). Classification of GBA variants and their effects in Synucleinopathies. Mov. Disord. 34, 1581–1582. doi: 10.1002/mds.27803 31769092

[ref16] Hall S. Surova Y. Öhrfelt A. Zetterberg H. Lindqvist D. Hansson O. (2015). CSF biomarkers and clinical progression of Parkinson disease. Neurology 84, 57–63. doi: 10.1212/WNL.0000000000001098, PMID: 25411441 PMC4336091

[ref17] Hoogland J. Boel J. A. De Bie R. M. A. Schmand B. A. Geskus R. B. Dalrymple-Alford J. C.. (2019). Risk of Parkinson's disease dementia related to level I MDS PD-MCI. Mov. Disord. 34, 430–435. doi: 10.1002/mds.27617, PMID: 30653248

[ref18] Hu M. T. M. Szewczyk-Królikowski K. Tomlinson P. Nithi K. Rolinski M. Murray C.. (2014). Predictors of cognitive impairment in an early stage Parkinson's disease cohort. Mov. Disord. 29, 351–359. doi: 10.1002/mds.25748, PMID: 24395708 PMC4235340

[ref19] Iwaki H. Blauwendraat C. Leonard H. L. Kim J. J. Liu G. Maple-Grødem J.. (2019). Genomewide association study of Parkinson's disease clinical biomarkers in 12 longitudinal patients' cohorts. Mov. Disord. 34, 1839–1850. doi: 10.1002/mds.27845, PMID: 31505070 PMC7017876

[ref20] Johar I. Mollenhauer B. Aarsland D. (2017). Cerebrospinal fluid biomarkers of cognitive decline in Parkinson's disease. Int. Rev. Neurobiol. 132, 275–294. doi: 10.1016/bs.irn.2016.12.001 28554411

[ref21] Jones J. D. Kuhn T. P. Szymkowicz S. M. (2018). Reverters from PD-MCI to cognitively intact are at risk for future cognitive impairment: analysis of the PPMI cohort. Parkinsonism Relat. Disord. 47, 3–7. doi: 10.1016/j.parkreldis.2017.12.006, PMID: 29233608 PMC5803409

[ref22] Leta V. Urso D. Batzu L. Weintraub D. Titova N. Aarsland D.. (2021). Constipation is associated with development of cognitive impairment in de novo Parkinson's disease: a longitudinal analysis of two international cohorts. J. Parkinsons Dis. 11, 1209–1219. doi: 10.3233/JPD-212570, PMID: 33843697

[ref23] Litvan I. Goldman J. G. Tröster A. I. Schmand B. A. Weintraub D. Petersen R. C.. (2012). Diagnostic criteria for mild cognitive impairment in Parkinson's disease: Movement Disorder Society task force guidelines. Mov. Disord. 27, 349–356. doi: 10.1002/mds.24893, PMID: 22275317 PMC3641655

[ref24] Liu G. Boot B. Locascio J. J. Jansen I. E. Winder-Rhodes S. Eberly S.. (2016). Specifically neuropathic Gaucher's mutations accelerate cognitive decline in Parkinson's. Ann. Neurol. 80, 674–685. doi: 10.1002/ana.24781, PMID: 27717005 PMC5244667

[ref25] Liu C. Cholerton B. Shi M. Ginghina C. Cain K. C. Auinger P.. (2015). CSF tau and tau/Aβ42 predict cognitive decline in Parkinson's disease. Parkinsonism Relat. Disord. 21, 271–276. doi: 10.1016/j.parkreldis.2014.12.027, PMID: 25596881 PMC4603566

[ref26] Liu G. Locascio J. J. Corvol J.-C. Boot B. Liao Z. Page K.. (2017). Prediction of cognition in Parkinson's disease with a clinical-genetic score: a longitudinal analysis of nine cohorts. Lancet. Neurol. 16, 620–629. doi: 10.1016/S1474-4422(17)30122-9, PMID: 28629879 PMC5761650

[ref27] Marinus J. Zhu K. Marras C. Aarsland D. Van Hilten J. J. (2018). Risk factors for non-motor symptoms in Parkinson's disease. Lancet. Neurol. 17, 559–568. doi: 10.1016/S1474-4422(18)30127-3 29699914

[ref28] Mata I. F. Leverenz J. B. Weintraub D. Trojanowski J. Q. Hurtig H. I. Van Deerlin V. M.. (2014). APOE, MAPT, and SNCA genes and cognitive performance in Parkinson disease. JAMA Neurol. 71, 1405–1412. doi: 10.1001/jamaneurol.2014.1455, PMID: 25178429 PMC4227942

[ref29] Mollenhauer B. Zimmermann J. Sixel-Döring F. Focke N. K. Wicke T. Ebentheuer J.. (2019). Baseline predictors for progression 4 years after Parkinson's disease diagnosis in the De novo Parkinson cohort (DeNoPa). Mov. Disord. 34, 67–77. doi: 10.1002/mds.27492, PMID: 30468694

[ref30] Nicoletti A. Luca A. Baschi R. Cicero C. E. Mostile G. Davì M.. (2021). Vascular risk factors, white matter lesions and cognitive impairment in Parkinson's disease: the PACOS longitudinal study. J. Neurol. 268, 549–558. doi: 10.1007/s00415-020-10189-8, PMID: 32865628 PMC7880923

[ref31] Norcliffe-Kaufmann L. Kaufmann H. Palma J.-A. Shibao C. A. Biaggioni I. Peltier A. C.. (2018). Orthostatic heart rate changes in patients with autonomic failure caused by neurodegenerative synucleinopathies. Ann. Neurol. 83, 522–531. doi: 10.1002/ana.25170, PMID: 29405350 PMC5867255

[ref32] Pedersen K. F. Larsen J. P. Tysnes O.-B. Alves G. (2017). Natural course of mild cognitive impairment in Parkinson disease: a 5-year population-based study. Neurology 88, 767–774. doi: 10.1212/WNL.0000000000003634, PMID: 28108638

[ref33] Pellecchia M. T. Savastano R. Moccia M. Picillo M. Siano P. Erro R.. (2016). Lower serum uric acid is associated with mild cognitive impairment in early Parkinson's disease: a 4-year follow-up study. J. Neural Transm. (Vienna, Austria: 1996) 123, 1399–1402. doi: 10.1007/s00702-016-1622-6 27682634

[ref34] Phongpreecha T. Cholerton B. Mata I. F. Zabetian C. P. Poston K. L. Aghaeepour N.. (2020). Multivariate prediction of dementia in Parkinson's disease. NPJ Parkinson's Dis. 6:20. doi: 10.1038/s41531-020-00121-2, PMID: 32885039 PMC7447766

[ref35] Poewe W. Seppi K. Tanner C. M. Halliday G. M. Brundin P. Volkmann J.. (2017). Parkinson disease. Nat. Rev. Dis. Primers. 3:17013. doi: 10.1038/nrdp.2017.13 28332488

[ref36] Real R. Martinez-Carrasco A. Reynolds R. H. Lawton M. A. Tan M. M. X. Shoai M.. (2022). Association between the LRP1B and APOE loci in the development of Parkinson's disease dementia. Brain: a. J. Neurol. doi: 10.1093/brain/awac414. [Epub ahead of print]. PMC10151192 36348503

[ref37] Schrag A. Siddiqui U. F. Anastasiou Z. Weintraub D. Schott J. M. (2017). Clinical variables and biomarkers in prediction of cognitive impairment in patients with newly diagnosed Parkinson's disease: a cohort study. Lancet. Neurol. 16, 66–75. doi: 10.1016/S1474-4422(16)30328-3, PMID: 27866858 PMC5377592

[ref38] Seifar F. Dinasarapu A. R. Jinnah H. A. (2022). Uric acid in Parkinson's disease: what is the connection? Mov. Disord. 37, 2173–2183. doi: 10.1002/mds.29209, PMID: 36056888 PMC9669180

[ref39] Stebbins G. T. Goetz C. G. Burn D. J. Jankovic J. Khoo T. K. Tilley B. C. (2013). How to identify tremor dominant and postural instability/gait difficulty groups with the movement disorder society unified Parkinson's disease rating scale: comparison with the unified Parkinson's disease rating scale. Mov. Disord. 28, 668–670. doi: 10.1002/mds.25383, PMID: 23408503

[ref40] Tan M. M. X. Lawton M. A. Jabbari E. Reynolds R. H. Iwaki H. Blauwendraat C.. (2021). Genome-wide association studies of cognitive and motor progression in Parkinson's disease. Mov. Disord. 36, 424–433. doi: 10.1002/mds.28342, PMID: 33111402 PMC9053517

[ref41] Terrelonge M. Marder K. S. Weintraub D. Alcalay R. N. (2016). CSF β-amyloid 1-42 predicts progression to cognitive impairment in newly diagnosed Parkinson disease. J. Mol. Neurosci. 58, 88–92. doi: 10.1007/s12031-015-0647-x, PMID: 26330275 PMC4738011

